# Attention-Based Scene Text Detection on Dual Feature Fusion

**DOI:** 10.3390/s22239072

**Published:** 2022-11-23

**Authors:** Yuze Li, Wushour Silamu, Zhenchao Wang, Miaomiao Xu

**Affiliations:** Xinjiang Multilingual Information Technology Laboratory, Xinjiang Multilingual Information Technology Research Center, College of Information Science and Engineering, Xinjiang University, Urumqi 830017, China

**Keywords:** scene text detection, feature pyramid network, spatial attention, multi-scale feature fusion, differentiable binarization

## Abstract

The segmentation-based scene text detection algorithm has advantages in scene text detection scenarios with arbitrary shape and extreme aspect ratio, depending on its pixel-level description and fine post-processing. However, the insufficient use of semantic and spatial information in the network limits the classification and positioning capabilities of the network. Existing scene text detection methods have the problem of losing important feature information in the process of extracting features from each network layer. To solve this problem, the Attention-based Dual Feature Fusion Model (ADFM) is proposed. The Bi-directional Feature Fusion Pyramid Module (BFM) first adds stronger semantic information to the higher-resolution feature maps through a top-down process and then reduces the aliasing effects generated by the previous process through a bottom-up process to enhance the representation of multi-scale text semantic information. Meanwhile, a position-sensitive Spatial Attention Module (SAM) is introduced in the intermediate process of two-stage feature fusion. It focuses on the one feature map with the highest resolution and strongest semantic features generated in the top-down process and weighs the spatial position weight by the relevance of text features, thus improving the sensitivity of the text detection network to text regions. The effectiveness of each module of ADFM was verified by ablation experiments and the model was compared with recent scene text detection methods on several publicly available datasets.

## 1. Introduction

With the rapid development and evolution of key technologies in the information age, traditional text recognition technologies have begun to be proposed, of which the representative Optical Character Recognition (OCR) technology is the conversion of characters in pictures or documents containing optical characters into character format. As the demands of real-life application scenes become more complex, more and more domains need to make use of the textual information contained in natural scenes. As the most direct representation of high-level semantic information, scene text plays an essential role in image understanding and is in demand for a wide range of applications.

Unlike traditional optical character recognition techniques, the complex background of scene images, uneven illumination, low light contrast, diverse text, and the distortion of image text perspective caused by photography make mature optical character recognition techniques not applicable to scene text recognition [1]. Nowadays, scene text recognition has become an important research direction in the field of computer vision, and scene text detection is used as a pre-procedure to determine whether the text is present in a scene image, and then locate the location of the text.

With the rapid development of deep learning technology, deep learning methods have achieved remarkable results in text detection tasks, and the existing convolutional neural networks already have good representation capabilities. At this stage, scene text detection technology has entered the era of deep learning, and the scene text detection methods based on deep learning can be summarized as follows.

The regression-based approach is inspired by general object detection methods. In early scene text detection studies, word-level or line-level ground truth is usually used to treat text as a particular kind of object, i.e., an object in object detection, and text objects are usually expressed in the form of rectangular boxes or quadrangular boxes with a specific orientation. The object detection algorithm is adapted to the text detection task by migrating and improving it. Although such methods are fast in detection and can avoid the accumulation of multi-stage mistakes, most existing regression-based methods cannot accurately and effectively solve the text detection problem due to the limitations of the text expression form (axial rectangle, rotated rectangle, or quadrilateral); especially, there are limitations in detecting long text, curved text or arbitrarily shaped text [2].

The segmentation-based approach, inspired by the idea of semantic segmentation, treats text detection as a classification problem that distinguishes between text and context. This approach locates text instances mainly by pixel classification. First, the text area is segmented from the scene, and then an additional post-processing step is applied to obtain the final text bounding box. The focus of research has also shifted from horizontal text to multi-directional text and more challenging arbitrarily shaped text, such as curved text. Although such methods are more suitable for arbitrarily shaped text detection, they are still likely to produce missed detections and false positives in the case of close line distance, text overlapping and text nesting.

It can be seen that most of the mainstream scene text detection methods have their respective strengths and weaknesses. The regression-based text detection results may have white space, but the miss detection rate is low for small-scale text, and the segmentation-based method can effectively solve the white space problem, but the discrimination of adjacent or multi-scale text is low. Overall, the segmentation-based text detection method is one of the most popular detection methods in recent years, and its segmentation results can more intuitively describe the text of arbitrarily shaped scenes. However, the lack of semantic and spatial information in the network limits the classification and localization capabilities of the network, and existing scene text detection methods suffer from the problem of losing important feature information in the process of extracting features from each layer. Despite the advantages of segmentation-based approaches in detecting arbitrarily shaped text, the lack of sufficient contextual information can also lead to false positives or missed detections. For example, although the Feature Pyramid Network (FPN) [3] expands the receptive field of the feature extraction network and can fuse features at different scales, the high-level semantic information of small-scale text is easily lost at the top of the network, leading to the weak detection capability of the model for multi-scale text.

To address the above problems, an Attention-based Dual Feature Fusion Model (ADFM) is proposed in this paper based on the lightweight network ResNet-18 to make the scene text detection network fuse and retain as much valid information as possible in the feature extraction process. In this model, we address the situation that feature extraction networks tend to lose low-level small-scale information in the feature extraction process and use FPN to compensate for the low-level details lost by high-level features while expanding the receptive field. At the same time, to reduce the interference and confounding effect of low-level semantic information on key text information, we use Bi-directional Feature Fusion Pyramid Module (BFM) to improve the characterization ability to acquire contextual semantic information and add the location-sensitive Spatial Attention Module (SAM) to the BFM structure. By combining location information that may be used as text regions, it is possible to directly and accurately locate text regions that are largely non-controversial and accurately detect the location of such text in natural scenes. Finally, the Differentiable Binarization (DB) process is introduced into the training process of the model, and the binarization threshold is set adaptively. The probability map generated by the segmentation method is transformed into text regions to achieve efficient and accurate text detection. 

The main contributions of this paper are as follows:

(1) We propose an attention-based bi-directional feature fusion pyramid model. This is a more efficient approach than previous methods to make up for the shortcomings of lightweight networks ResNet-18 in feature extraction and reduce missed and false detections caused by large differences in text scale, which trades a small increase in computation for better accuracy based on a lightweight and efficient backbone network.
(2) Position-sensitive spatial attention is introduced, focusing on the intermediate features of two-stage feature fusion. Unlike previous methods of attention weighting on multi-scale feature maps, our proposed method focuses on the one feature map with the highest resolution and strongest semantic features in the FPN, which results in better spatial attention weighting and can accommodate both strong and weak responses of texts at different scales.
(3) Experiments on the multi-directional English text dataset ICDAR2015 [4], Chinese dataset CTW1500 [5] and long text dataset MSRA-TD500 [6] demonstrate the effectiveness of the proposed model. The model not only ensures the quality of feature extraction but also achieves a good balance between speed and accuracy due to the lightweight network.

The rest of this paper is organized as follows. In Section 2, we briefly introduce the related work, which describes the regression-based scene text detection method and the segmentation-based scene text detection method, respectively. Section 3 describes the proposed method in this paper in detail. Section 4 shows the results of ablation experiments and comparison experiments, verifies the effectiveness of each module in our proposed method and its superiority compared to most mainstream methods, and presents the visualization results for various contexts. Section 5 summarizes the work we have conducted and puts forward the prospects.

## 2. Related Work

### 2.1. Scene Text Detection

Early approaches to scene text detection typically detected individual characters or components and then grouped them into words. Deep learning-based scene text detection methods can be classified into two categories: regression-based methods and segmentation-based methods.

Regression-based algorithms for scene text detection were mainly inspired by Faster R-CNN [7], Mask R-CNN [8], SSD [9] and other algorithms, which are direct regression methods for text instance bounding boxes. Specifically, a convolutional neural network is used to adjust the size and position of the anchor box during the regression to accurately locate the text by determining whether the preset text box overlaps with the ground truth of the text area. Based on Faster R-CNN, RRPN [10] developed a rotated region proposal network to detect text, using a rotated bounding box containing angle parameters, adjusting the angle information in training and performing bounding box regression to improve the accuracy of inclined text detection. TextBoxes [11] directly modifies the anchoring scale and shape of the SSD convolution kernel to detect text with extreme aspect ratios. TextBoxes++ [12] further regresses quads instead of horizontal bounding boxes for multi-directional text detection. RRD [13] applies rotation invariant and sensitive features for text classification and regression for better long-text detection from two independent branches. SSTD [14] generates textual attention maps, enhances textual regions of feature maps, suppresses background information and facilitates tiny text. Mask Text Spotter [15] and SPCNet [16] treat text detection as instance segmentation and use Mask R-CNN for arbitrary text detection. Regression-based methods usually have simple post-processing algorithms (e.g., non-maximal suppression). However, most existing regression-based methods rely on preset text boxes and are not good at determining bounding boxes for irregularly shaped text. The orientation, size and aspect ratio of text in natural scenes vary widely. To make the pre-defined text boxes overlap with the text area, many methods add text boxes with different orientations, sizes and aspect ratios, but this increases the complexity and computational effort of the model.

Segmentation-based approaches usually utilize semantic segmentation combined with pixel-level prediction and post-processing algorithms to obtain bounding boxes. All pixels within the text bounding box are treated as positive sample regions. Different representations are used to describe the text regions and then the text instances are reconstructed by specific post-processing. The advantage of these methods is the ability to extract the text of arbitrary shapes. PixelLink [17] processes text or non-text link predictions at the pixel level, then post-processes to obtain the text box and exclude noise. EAST [18] predicts the distance from the center point to the four edges and the rotation angle of the text box directly from the Rotated Box. By predicting the four vertices of irregular quadrilateral by QUAD, we can directly infer the pixel-level quadrilateral of candidate words without anchor mechanism and proposal detection. TextSnake [19] models text instances using text centerlines, which are capable of representing text in arbitrary shapes. PSENet [20] uses FCN to predict multi-scale text instances and then uses width-first traversal to expand the text kernel and reconstruct the text instances. PAN [21] enhances the feature extraction capability of the network by using layer stacking. The multi-scale fused feature maps are delivered to the segmentation network to predict the text regions. Finally, post-processing of pixel aggregation is used to further refine the bounding boxes of text regions. DBNet [22] uses continuously differentiable binarization functions instead of discrete binaries, which are inserted into the segmentation network for joint optimization. The binarization part can be trained and tuned with the training process. Robust binarization thresholds are obtained by adaptively learning for each pixel during the optimization process. The post-processing process is simplified, and the efficiency and accuracy of text detection are improved. However, most segmentation-based methods employ heavy frameworks or complex pipelines, which usually reduce their inference speed. Our approach uses a lightweight network while compensating for its shortcomings in feature extraction capability and receptive field, balancing the accuracy and efficiency of text detection.

### 2.2. Multi-Scale Feature Fusion

One challenge of object detection is multi-scale target detection. Text detection, as special object detection, faces almost the same problem. Convolutional neural networks perform feature extraction layer by layer. The higher-layer network has a larger receptive field and strong representation of semantic information, but the feature map has low resolution and lacks spatial feature details. Multiscale feature fusion FCN [23] proposed the first fully convolutional network with an up-sampling layer to fuse multi-scale features. U-net [24] inherited the structure of FCN while using jump connection which directly connects low-level features and high-level features. FPN uses top-down and lateral connectivity based on the SSD bottom-up approach to add low-level semantic features. PSPNet [25] and Deeplabv3 [26] propose the Pyramid Pool Module (PPM) and Atrous Space Pyramid Pool (ASPP) for multi-scale feature fusion, respectively. The authors of [27] extracted local spatial texture features using a local binary image feature extraction method to construct a multi-feature vector. Furthermore, ref. [28] processes an ensemble classification algorithm is operated to train a multi-label classifier in a new feature space. Based on [29], ref. [30] proposed a three-phase large-scale optimizer. The BFM we proposed achieves a two-stage enhancement of the FPN, which weakens the interference and confounding effect of the low-level semantic information on the key text information, and smoothly fuses the high-level and low-level semantic information.

### 2.3. Attention Mechanism

Some existing image classification methods use channel attention and spatial attention to improve the accuracy of image classification. CBAM [31] inferred attention weights sequentially along two dimensions, spatial and channel. By multiplying the attention with the original feature map, weights are assigned to the feature map so that the features complete adaptive adjustment. CCNet [32] proposes iterative crossover attention to obtain more effective object features by computing the interrelationships between the object feature pixels and all other pixels in the feature map and weighting the features of the object pixel with such interrelationships. In this way, more effective object features are obtained. DANet [33] uses dual attention networks to simulate semantic interdependencies in space and channel dimensions, adaptively integrating local features and global dependencies. These methods focus on portraying independent features of different dimensions with different types of attention. The focus of the SAM we proposed is to highlight all possible location information in the feature space that could be text regions.

## 3. Methodology

In this section, we will introduce ADFM and its two sub-modules BFM and SAM at first. Then, we will present a simplified post-processing procedure for generating binary maps using DB.

**Figure 1:**
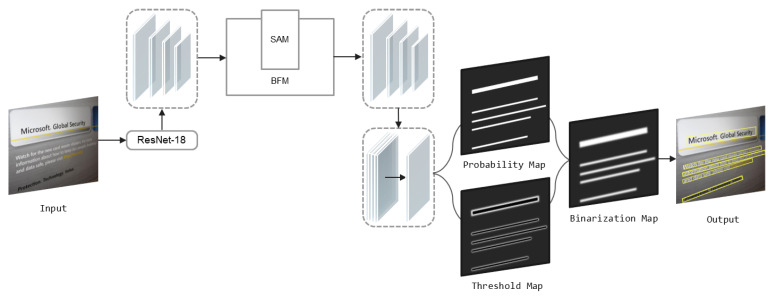
It shows the overall framework of ADFM. Firstly, a lightweight backbone network is used. Secondly, two more efficient feature fusion modules named BFM and SAM are designed to improve the network feature extraction. Finally, DB is introduced to improve the tedious inference process.

The architecture of our proposed model is shown in Figure 1.

First, to improve efficiency, we use the lightweight model ResNet-18 as the backbone network. However, lightweight backbones produce features that tend to have smaller receptive fields and weaker representation capabilities. For this problem, we propose BFM. The output of the backbone network is fed into the BFM, and the features can be effectively refined. Second, the intermediate features generated in the up-scale enhancement stage on this module are passed into the spatial attention module SAM to generate location-sensitive spatial contextual features f. Input f into the down-scale enhancement stage. Then, the multi-scale feature maps output from the down-scale enhancement stage is unified to the same scale by up-sampling. Feature fusion is then performed. The Probability Map (P) and the Threshold Map (T) are predicted by the final obtained feature map F. Then, the approximated Binarization Map (B) is calculated using P and F. During the training period, supervising the P, T and B. The text bounding box can be subsequently obtained from the B or P.

### 3.1. Bi-Directional Feature Fusion Pyramid Module (BFM)

**Figure 2:**
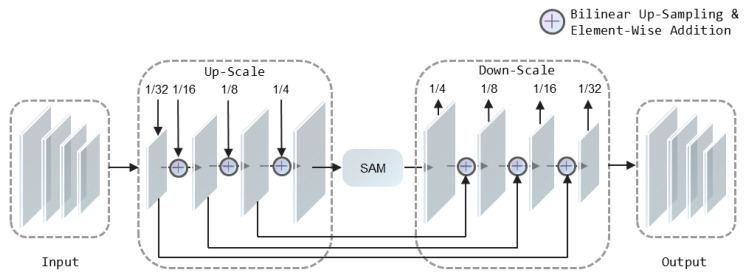
It shows the details of BFM. The input to this module is four-scale feature maps extracted by the backbone network starting from Conv2. The output is the enhanced multi-scale feature maps. “+” is the element-wise addition after up-sampling to the same scale.

BFM is a two-stage feature fusion module, as shown in Figure 2.

Similar to FPN, BFM can enhance features at different scales by fusing low-level and high-level information, thus expanding the field of reception. It includes two stages up-scale enhancement and down-scale enhancement. The first stage enhances the contribution of low-level semantic information in feature extraction. The second stage weakens the interference and confounding effect of low-level semantic information on key text information.

The four feature maps generated by the backbone network starting from Conv2 have a resolution of 1/4, 1/8, 1/16 and 1/32 compared to the input image. The up-scale enhancement acts on the feature pyramids extracted by the backbone network. In this stage, the enhancement is iterated over the feature map in the stride of 1/32 to 1/4. The down-scale enhancement acts on the feature pyramids generated in the up-scale enhancement stage in a stride of 1/4 to 1/32. The feature pyramid output from the down-scale enhancement stage contains feature maps at different scales. It is necessary to unify several feature maps of different scales into the same scale by up-sampling first. The fused feature map is then output to participate in prediction at the next stage.

### 3.2. Spatial Attention Module (SAM)

The feature maps at different scales have different perceptual information, so they focus on describing text instances at different scales separately. For example, low-level feature maps can perceive the details of small text instances, but cannot capture the whole of large text instances. Additionally, the high-level feature map captures the global information of large text instances while ignoring the details of small text instances. 

**Figure 3:**
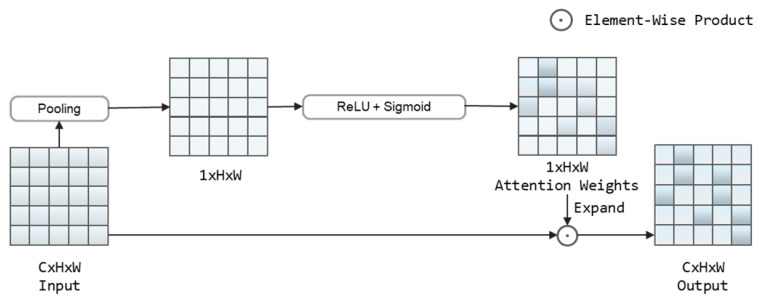
It shows the details of SAM. Spatial average pooling, Conv-ReLU and Conv-Sigmoid are used sequentially for the input feature map to obtain the attention weights. The attention weights are assigned to the input feature map through element-wise production.

We added more details contained in the low-level feature maps to the high-level feature maps through the up-scale enhancement stage. To fully utilize and strengthen the features obtained in the up-scale enhancement stage, we use a spatial attention module that acts on the lowest-level feature map obtained from the up-scale enhancement and focuses on the location information related to the text objects. The sensitivity of the text detection network to text regions is improved. SAM is shown in Figure 3.

As shown in Figure 3, applying spatial average pooling to the input feature map $X\in R^{C\times H\times W}$ to normalize the channel *C* and obtain $X_{1}\in R^{1\times H\times W}$.

(1) $$X_{1}=P_{avg}\left(X\right)$$

Conv-ReLU and Conv-Sigmoid operations are then performed on $X_{1}$ to calculate the attention weights $A\in R^{1\times H\times W}$.

(2) $$A=Sigmoid\left(Conv\left(Relu\left(Conv\left(X_{1}\right)\right)\right)\right)$$

After expanding the attention weight *A* to $A\in R^{C\times H\times W}$, then *A* product with the input feature map *X*. The attention weight is assigned to *X* to obtain the feature $F\in R^{C\times H\times W}$ that is sensitive to location information.

(3) F=X⊙Expand(A)

where Expand( ) denotes the dimensional expansion and ⊙ denotes the element-wise product.

Finally, the attention-guided feature *F* is fed into the down-scale enhancement stage of BFM.

### 3.3. Differentiable Binarization (DB)

#### 3.3.1. Binarization

Differentiable binarization is an approximate step function to model the standard binarization function. This allows it to be optimized with the segmentation network during training. The differentiable binarization with a self-adaptive threshold can not only distinguish text regions from the background, but also separate closely connected text instances.

(4) $${\hat{B}}_{i,j}=\frac{1}{1+e^{-k\left(P_{i,j}-T_{i,j}\right)}}$$

The above equation *T* represents the threshold map for network learning, *k* is a factor, and the output $\hat{B}$ represents the approximate binarization map. The differentiable binarization makes the model focus on optimizing the prediction of ambiguous regions. Moreover, the Sigmoid function alleviates the problem of “The least upper bound and the greatest lower bound” and DB further decreases the penalty for extremely small or large values.

#### 3.3.2. Label Generation

The binarization map and the probability map use the same labels. The following equation defines an offset and then shrinks each labeled box by such an offset, resulting in a labeled graph called $G_{s}$, with the original labeled text box as *G*.

(5) $$D=\frac{A\left(1-r^{2}\right)}{L}$$

where *r* is the pre-set scaling factor, *L* is the perimeter of the label box, and *A* is the area of the label box. The label generation process of the threshold map is as follows: first, the text polygon *G* is expanded by *D* to get $G_{d}$. The labels of the threshold map are generated by calculating the normalized distance from the pixel between the $G_{d}$ box and the $G_{s}$ box to the nearest edge of the *G* box. Since the label of the threshold map cannot be 1 or 0, the label values need to be rescaled by reducing 1 to 0.7 and enlarging 0 to 0.3.

#### 3.3.3. Loss Function

The $L_{1}$ loss function and the binary cross entropy (BCE) loss function is used here to optimize our network. The formula for the loss function is as follows:

(6) $$L=L_{s}+\alpha \times L_{b}+\beta \times L_{t}$$

where $L_{t}$ is the loss of the threshold map, $L_{s}$ is the loss of the probability map and $L_{b}$ is the loss of the binarization map, where α and β take the values of 1.0 and 10, respectively. Solving for $L_{s}$ and $L_{b}$ using BCE:

(7) $$L_{s}=L_{b}=\underset{i\in S_{i}}{\sum }y_{i}\log_{x_{i}}+\left(1-y_{i}\right)\log\left(1-x_{i}\right)$$

The ratio of positive samples to negative samples is 1:3. $L_{t}$ is using $L_{1}$ loss:

(8) $$L_{t}=\underset{i\in R_{d}}{\sum }|y_{i}{}^{*}-x_{i}{}^{*}|$$

where $y_{i}{}^{*}$ is the label of the threshold map and $R_{d}$ is all the pixel in $G_{d}$. 

In the inference phase, text regions can be generated from the probability map or the approximate binarization map, and it is more efficient to use the probability map. The process of generating a text region using a probability map is to first obtain a binarization map using a threshold of 0.2 for the probability map. Then use the binarization map obtained in the previous step to calculate the connected area of the text, and finally use the offset $D^{\prime }$ to enlarge the connected area to obtain the text area. $D^{\prime }$ is calculated as follows:

(9) $$D^{\prime }=A^{\prime }\times r^{\prime }L^{\prime }$$

where $r^{\prime }$ is set to 1.5, $A^{\prime }$ represents the area of the connected area, and $L^{\prime }$ is the perimeter of the connected area.

## 4. Experiments

To verify the practicability and efficiency of the proposed method, we conducted ablation experiments for each of the key modules to check the impact of each module on the model performance. Then, comparison experiments are conducted on three public datasets to evaluate the algorithm using the commonly used text detection metrics Precision (*P*), Recall (*R*) and F-measure (*F*). The number of real text boxes predicted as text boxes are recorded as *TP*, the number of real text boxes predicted as background areas are recorded as *FP*, the number of background areas predicted as text boxes are recorded as *FN* and the number of background areas predicted as the background is recorded as TN, then the calculation formula of comprehensive values of *P*, *R* and *F* are as follows:

(10) $$P=\frac{TP}{TP+FP}$$

(11) $$R=\frac{TP}{TP+FN}$$

(12) $$F=\frac{2PR}{P+R}$$

### 4.1. Datasets and Experimental Environment

For model training, we pre-train our model using the SynthText [34] dataset, which is a synthetic dataset consisting of 800 k images that are synthesized from 8k background images. This dataset is only used to pre-train our model. In the part of model fine-tuning and validation, we use the ICDAR2015, CTW1500 and MSRA-TD500 datasets to validate the algorithm performance and conduct comparison experiments.

The ICDAR2015 dataset contains 1500 images, including 1000 images in the train set and 500 images in the test set. The image backgrounds are complex, the text scales vary greatly, and the images are blurred, from which all English text examples are labeled at the word level using quadrilateral boxes.

The CTW1500 dataset has 1000 train images and 500 test images and is a widely used dataset for arbitrarily shaped text detection, which contains long text lines, and images are labeled at the text line level.

The MSRA-TD500 dataset is small in size, containing only 500 natural scene images, of which 300 are used as the train set and 200 as the test set. The text languages include Chinese and English, all of which are labeled at the text line level, and the text scales are highly variable and the text is arbitrarily oriented.

We trained our model on two NVIDIA Tesla V100 16G GPUs. The CUDA version is 11.6. For training, the model is fine-tuned by 1500 epochs on the real dataset using a network that has been pre-trained on the synthetic dataset SynthText. The initial learning rate is set to 0.001, using the Adam [35] optimizer. The batch size is set to 8. The training data are enhanced by random rotation angles, cropping and flipping in the range of (−10, 10), and all images are resized to 640×640. 

Since test images of different scales have a large impact on the detection result, the aspect ratio of the test images is maintained in the inference stage and the input images are resized by setting the appropriate short edge size for each dataset.

### 4.2. Ablation Experiments

To verify the validity of each part of the ADFM model separately, ablation experiments were conducted on the public dataset ICDAR2015 for the model. The results of the ablation experiments are shown in Table 1.

Our ablation experiments verified the effectiveness of the independent and pluggable modules in groups 0–3. Specifically, the effectiveness of SAM was verified individually through groups 1 and 2, and the effectiveness of DB was verified individually through groups 1 and 3. Based on the time overhead and nested structure considerations, the ablation experiments for BFM are carried out in a composite module. Since the BFM is embedded with SAM, it needs to be split within the structure for experiments. Therefore, we conducted ablation experiments inside the structure for the main improved BFM and SAM parts based on ensuring the application of DB through groups 3–6 and verified the effectiveness of BFM without SAM through groups 3 and 5, the effectiveness of SAM through groups 3 and 4, and the effectiveness of BFM with SAM embedded through groups 3 and 6.

It can be seen that on the ICDAR2015 dataset, compared to the base model, the introduction of SAM in the base model improved P by 1.1%, R by 3.0% and F by 2.8%; the introduction of BFM without SAM in the base model improved P by 0.9%, R by 5.0% and F by 3.3%; the introducing of BFM with SAM embedded in the base model, P improved by 1.5%, R improved by 4.9% and F improved by 3.5%;

Combining the enhancement effects on model accuracy and recall after each module is added, it is demonstrated that, based on the application of DB for the post-processing process, the introduction of BFM and SAM modules can optimize the feature extraction process of the model, which results in performance improvement for the whole model.

### 4.3. Comparison Experiments

To verify the efficiency of ADFM, it is compared with other methods on three public datasets, and the experiment results are shown in Table 2, Table 3 and Table 4. The best ADFM model pre-trained on the SynthText dataset is used to participate in the test.

On the ICDAR2015 dataset, compared to the base model, ADFM improved P by 1.4% to 90.2%, R by 6.4% and F by 4.4%, and performed better than most of the more advanced algorithms in recent years. Compared with the test results of DBNet trained with ResNet-18, the P improved by 3.4%, R by 0.3% and F by 1.8%; compared with the test results of the more recent algorithm DBNet++ [36] trained on the ResNet-18 backbone, the P is improved by 0.1%, the R is improved by 1.5% and the F is improved by 1%. The results of the comparison with other methods are shown in Table 2.

**Table 2:** Detection results on the ICDAR2015 dataset.

Method	P (%)	R (%)	F (%)
CTPN [37]	74.2	51.6	60.9
EAST	83.6	73.5	78.2
PAN	84	81.9	82.9
PSENet	86.9	84.5	85.7
SPCNet	88.7	85.8	87.2
CRAFT [38]	89.8	84.3	86.9
ContourNet [39]	86.1	87.6	86.9
DBNet (ResNet-18)	86.8	78.4	82.3
DBNet++(ResNet-18)	90.1	77.2	83.1
**Base (ResNet-18)**	**88.8**	**72.3**	**79.7**
**Ours (ResNet-18)**	**90.2**	**78.7**	**84.1**
PMTD [40]	91.3	87.4	89.3

On the MSRA-TD500 dataset, ADFM improved P by 1.6% to 87.9%, R by 0.7% and F by 1.1% compared to the base model, the precision was better than most of the more advanced algorithms in recent years. Compared with PAN and PixelLink, the P was improved by 3.5% and 4.9%, respectively; compared with TextSnake, the P was improved by 4.7%, the R was improved by 4.2%, and the F was improved by 4.4%; the P was comparable to the test results of the latest algorithm DBNet++ trained with ResNet-18. The results of the comparison with other methods are shown in Table 3.

**Table 3:** Detection results on the MSRA-TD500 dataset.

Method	P (%)	R (%)	F (%)
SegLink [41]	86	70	77
EAST	87.3	67.4	76.1
PAN	84.4	83.8	84.1
RRPN	82	68	74
CRAFT	88.2	78.2	82.9
TextSnake	83.2	73.9	78.3
PixelLink	83	73.2	77.8
**Base (ResNet-18)**	**86.3**	**77.4**	**81.6**
**Ours (ResNet-18)**	**87.9**	**78.1**	**82.7**
DBNet++(ResNet-18)	87.9	82.5	85.1

On the CTW1500 dataset, ADFM improved by 1.9% in P and 0.3% in F compared to the base model; compared with the test results of the superior algorithm DBNet trained with ResNet-18 in recent years, the P improved by 2.9%, the R improved by 0.8% and the F improved by 1.8%; compared with PSENet and PAN, the P was improved by 2.9% and 1.3%, respectively; compared with the test results of the more recent algorithm DBNet++ trained with ResNet-18, the P was improved by 1% to 87.7%. The results of the comparison with other methods are shown in Table 4.

**Table 4:** Detection results on the CTW1500 dataset.

Method	P (%)	R (%)	F (%)
EAST	78.7	49.1	60.4
PAN	86.4	81.2	83.7
PSENet	84.8	79.7	82.2
CRAFT	86	81.1	83.5
TextSnake	85.3	67.9	75.6
DBNet (ResNet-18)	84.8	77.5	81.0
ContourNet	84.1	83.7	83.9
**Base (ResNet-18)**	**85.8**	**77.2**	**81.3**
**Ours (ResNet-18)**	**87.7**	**78.3**	**82.8**
DBNet++(ResNet-18)	86.7	81.3	83.9

On the above three datasets, a comprehensive analysis of the experimental results comparing our model with existing methods shows that our model achieves better performance in three evaluation metrics compared with the base model. On the regular text dataset ICDAR2015, our model outperforms the latest model DBNet++ in three evaluation metrics and outperforms most existing models such as regression and segmentation-based two-stage hybrid method ContourNet and segmentation-based method PSENet, PAN and CRAFT in the precision rate of detection. On the multilingual dataset MSRA_TD500, which contains both Chinese and English, our model outperforms the segmentation-based methods TextSnake and PixelLink in three evaluation metrics. In terms of precision rate of detection, our model outperforms most existing models and achieves comparable precision rates with the top-performing model CRAFT and DBNet++. On the dataset CTW1500, which contains a large amount of arbitrarily shaped text and long text, our model outperforms the segmentation-based methods DBNet and TextSnake in three evaluation metrics and outperforms most existing models, such as the latest segmentation-based method DBNet++, and the regression and segmentation-based two-stage hybrid method ContourNet, in terms of precision rate of detection.

### 4.4. Visualization Results

**Figure 4:**
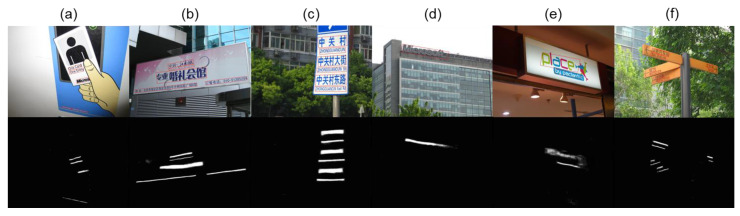
Visualization results. (**a**,**b**) are scene text detection under multi-scale variation, with significant variations in text size and stroke thickness in the same image. (**c**) is scene text detection of adjacent texts. (**d**) is scene text detection where the text is easily confused with the background. (**e**) is scene text detection of abstract art texts. (**f**) is scene text detection of multi-directional texts.

Scene text detection usually faces many challenges, such as complex backgrounds, text inclined in multiple directions, text in abstract art characters, uneven lighting and text containing multiple languages. Text texture is similar to the background and is therefore easily confused with the background, text with large scale variations, close text lines, etc. Figure 4 below shows the text detection results in some representative cases:

The above results show that ADFM predicts classification labels at the pixel level and can better adapt the text in various image conditions. We can see that our network performs well in natural scene text detection, especially with high precision, and can achieve better detection with a lightweight backbone network and smaller computational overhead. Experiments show that BFM and SAM are important for multi-scale text feature extraction and position information enhancement, which can improve the detection precision of the base model with comparable detection efficiency. In detail, the rich semantic information embedded in the multi-scale feature maps can effectively improve the classification ability of the network and capture details that are easily overlooked, the critical spatial information helps to enhance the detection network’s perception of the text, and the differentiable binarization method helps to determine more precise boundaries. Meanwhile, in various challenging scenes, such as text with large-scale variations, text with similar background texture, adjacent text with close line spacing, abstract fonts, multilingual and multi-directional text, etc. In particular, the model can effectively handle the drastic scale variations of text, improve the effectiveness of text detection in natural scenes, and detect the scene text accurately, which to some extent is inseparable from our proposed model.

## 5. Conclusions

In this paper, we propose a lightweight scene text detection model for arbitrary shapes, and improve the segmentation-based scene text detection method from two aspects:

(1) A location-sensitive spatial attention mechanism, SAM, is proposed, focusing on the intermediate features of the two-stage feature fusion, assigning corresponding location weights to them and highlighting the pixels that contain more significant text features.
(2) BFM is used instead of FPN, and SAM is introduced to the intermediate features of the two-stage feature fusion network of BFM, which reduces the interference of its confounding effect on the strong semantic features at the higher levels in the process of enhancing the weak semantic features at the lower levels. Both of these modules effectively improve the precision of text detection. Even with a lightweight backbone network ResNet-18, our approach can balance detection quality and speed, achieving good coordination between accuracy and efficiency measures.

We will further optimize the structure of the network based on the current model, reduce the complexity of the feature extraction network, balance quality and efficiency, and improve the overall performance of the algorithm. In the future, we will further investigate more universal scene text detection models in multi-lingual scenes.

## Figures and Tables

**Table 1:** Results of the ablation experiments on the ICDAR2015 dataset.

Backbone	BFM	SAM	DB	P (%)	R (%)	F (%)
ResNet-18				84.6	76.1	80.1
ResNet-18		√		85.3	76.3	80.6
ResNet-18			√	88.3	73.3	80.1
ResNet-18		√	√	89.4	77.3	82.9
ResNet-18	√		√	89.2	78.3	83.4
ResNet-18	√	√	√	89.8	78.2	83.6

## Data Availability

Not applicable.
